# Molecular characterization of *Vibrio* species isolated from dairy and water samples

**DOI:** 10.1038/s41598-023-42334-4

**Published:** 2023-09-16

**Authors:** Mona A. El-Zamkan, Ahmed Shaban Ahmed, Hanan H. Abdelhafeez, Hams M. A. Mohamed

**Affiliations:** 1 Department of Food Hygiene and Control (Milk Hygiene), Faculty of Veterinary Medicine, South Valley University, Qena, 83523 Egypt; 2 Department of Cell and Tissues, Faculty of Veterinary Medicine, Assiut University, Assiut, 71526 Egypt; 3 Department of Microbiology, Faculty of Veterinary Medicine, South Valley University, Qena, 83523 Egypt

**Keywords:** Microbiology, Molecular biology

## Abstract

*Vibrio* species can cause foodborne infections and lead to serious gastrointestinal illnesses. The purpose of this research was to detect the *Vibrio cholerae* and *Vibrio parahaemolyticus* in raw milk, dairy products, and water samples. Also, it investigated the virulence factors, antibiotic resistance and biofilm formation in isolated bacteria. Conventional and molecular approaches were used to identify the isolates in this study. *Vibrio* species were detected in 5% of the samples. *Vibrio cholerae* and *Vibrio parahaemolyticus* were isolated from 1.25 and 1.5%, respectively, of the total samples. Penicillin resistance was detected in all strains of *Vibrio cholerae* and *Vibrio parahaemolyticus*, with a MAR index ranging from 0.16 to 0.5. Four isolates were moderate biofilm producer and three of them were MDR. When *Vibrio cholerae* was screened for virulence genes, *ctx*AB, *hly*A, and *tcp*A were found in 80, 60, and 80% of isolates, respectively. However*, tdh* + /*trh* + associated-virulence genes were found in 33.3% of *Vibrio parahaemolyticus* isolates.

## Introduction

*Vibrio* species can cause the bacterial disease Vibriosis, which is a leading cause of mortality^1^. The gram-negative bacteria *Vibrio cholerae* (serogroups O1 and O139) also causes cholera epidemics^2^. Aquatic habitats frequently harbour non-O1/non-O139 strains, which have lately been connected to occasional instances of diarrhoea^3^. Ingestion of *V. parahaemolyticus*-contaminated food can cause nausea, diarrhoea, vomiting, and other food poisoning signs^4^. In extreme situations, it can result in sepsis and death^5^. *V. parahaemolyticus* food poisoning is becoming more common, that is dangerous to the health and safety of the general population. Its risk as a foodborne illness has overtaken that of salmonellosis, seriously compromising people's health and resulting in enormous economic losses^6^. Cholera toxin (CT), which is expressed by the *ctxAB* gene, and the toxin-coregulated pilus (TCP), which is encoded by the *tcpA* gene, are the two main virulence factors of *V. cholerae* O1 and O139^2^. While hemolytic toxin, which includes heat-resistant hemolytic toxin (TDH), hemolytic toxin linked with heat-resistant hemolytic toxin (TRH), and heat-labile hemolytic toxin, is the main factor contributing to *V. parahaemolyticus*'s pathogenicity (TLH)^7^.

Antibiotics are the most commonly used treatment for bacterial diseases, and most *Vibrio* spp. are susceptible to them. Antibiotics that are effective against Vibriosis include tetracycline, quinolones, trimethoprim, oxytetracycline potentiated sulfonamides, quinolones, oxolinic acid, and sarafloxacin^8,9^. Due to the widespread and disorderly use of antibiotics, resistant strains of *Vibrio* spp. are developing and becoming more prevalent^10^, It could be harmful to human health because it introduces resistant bacteria into the food chain or because mobile genetic elements can spread resistance genes to other human pathogens^11,12^.

*Vibrio* spp. are able to produce adhesion factors, allowing them to adhere to surfaces and cause the development of biofilms^2^. *Vibrio* spp. enter food products through contamination of equipment and instruments by growing biofilms on inert surfaces like stainless steel, polyvinyl chloride, and glass, which leads to cross-infection and considerable antibiotic resistance^6^. This renders disinfectants, antibodies, and antibiotics ineffective against bacteria in the biofilm mass^2^. So this study was directed to detect the *V*. *cholerae* and *V*. *parahaemolyticus* in raw milk, dairy products, and water samples. Also, investigation of *Vibrio* isolates for existence of virulence factors, antibiotic resistance and biofilm formation.

## Results

### Phenotypic identification of *Vibrio* spp.

Presumptive colonies of *Vibrio* spp. (yellow, blue-green) on TCBS agar plates were biochemically characterized. Out of 60 isolates, 33.3% (20/60) were presumptively selected as *Vibrio* spp. As Gram –ve curved rods exhibits ability for growth in 1% NaCl broth, oxidase positive reaction, has the ability to ferment glucose, nitrate reduction,H_2_S production, lysine decarboxylation and gelatin liquefaction inveterate the characteristics of isolates.

### Characterization of *V. cholerae *and *V. Parahaemolyticus* using *ctx* and *tox*R genes

**Figure 1:**
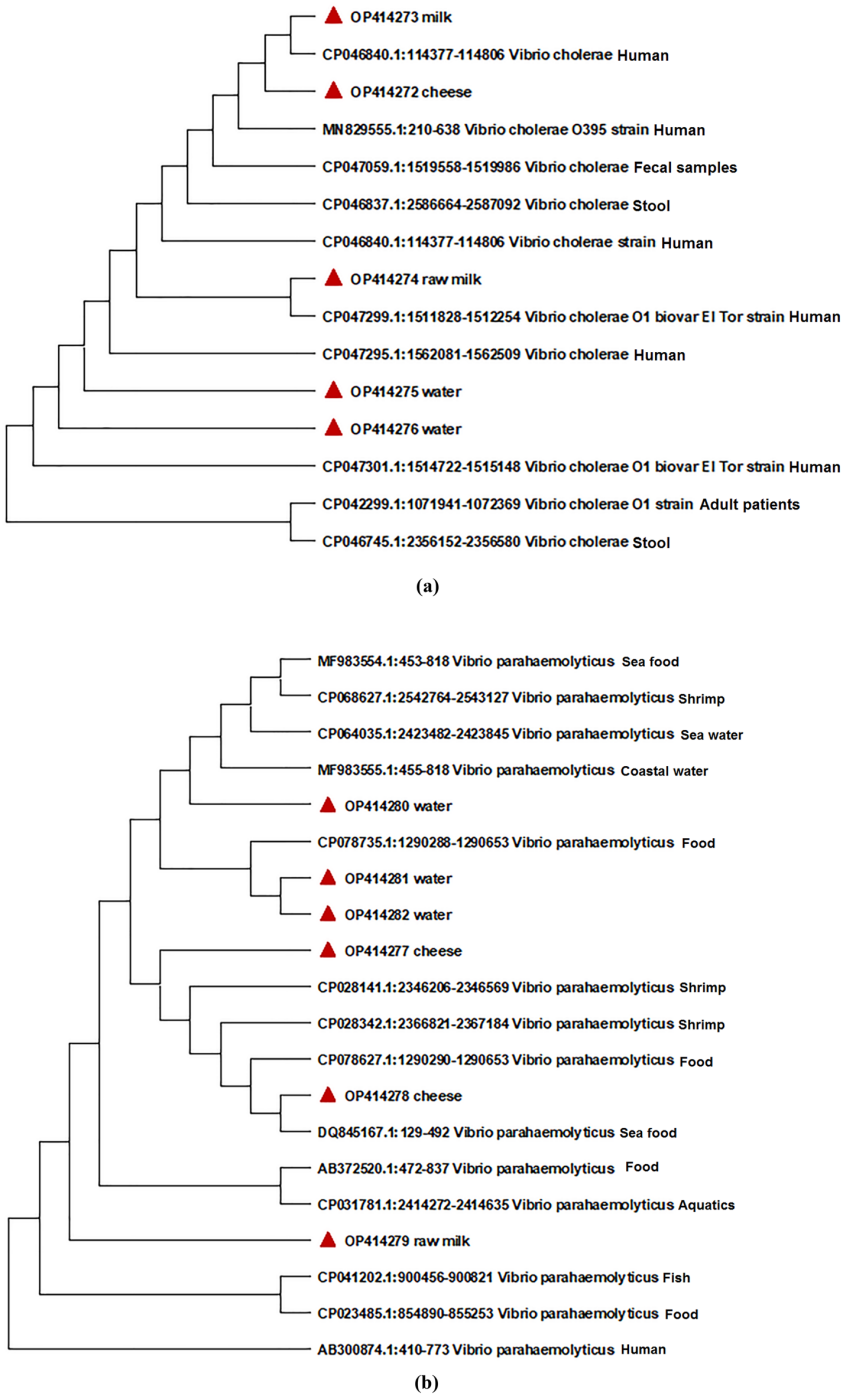
The Maximum Likelihood tree shows the *ctx* and *tox*R genes phylogenetic relationships of (**a**) *V. cholerae* and (**b**) *V. parahaemolyticus* isolated from raw milk, cheese and water and phylogenetically related reference strains on GenBank.

These isolates were molecularly confirmed as *Vibrio* spp. using housekeeping *16S rDNA gene primers* and recovered from 2 and 6% of dairy and water samples, respectively, and a total of 5% of the total samples. Eleven of the molecularly confirmed *Vibrio* spp., were later identified on species level by *ctx* and *tox*R amplification, sequencing, and phylogenetic analysis as *V. cholerae* (n = 5, 1.25%) and *V. parahaemolyticus* (n = 6, 1.5%) (Table 1, Fig. 1). The sequences obtained in this investigation have been put in GenBank under the accession Nos. (OP414272, OP414273, OP414274, OP414275, OP414276, OP414277, OP414278, OP414279, OP414280, OP414281 and OP414282).

**Table 1:** Prevalence of *V. cholerae* and *V. parahaemolyticus* isolated from the examined samples.

Type of samples	No. of samples	No. of *Vibrio* species positive samples (%)	No. of species (%)		
			*V. cholerae*	*V. parahaemolyticus*	Other *Vibrio* spp.
Raw milk	100	3 (3)	2 (2)	1(1)	0
Domiatti cheese	50	1 (2)	0	1 (2)	0
Kareish cheese	50	2 (4)	1 (2)	1 (2)	0
Yoghurt	50	0	0	0	0
Ice cream	50	0	0	0	0
Sub-Total	300	6 (2)	3(1)	3(1)	0
Water	100	14 (14)	2 (2%)	3(3%)	9 (9%)
Total	400	20 (5)	5 (1.25)	6 (1.5)	9 (2.25)

### Antimicrobial resistance profile, virulence and biofilm production of *V. cholerae *and *V. parahaemolyticus*

The antibiotic resistance profiles of *Vibrio* isolate for the twelve antibiotics are stated in Table 2. All the obtained isolates exhibited resistance to Penicillin family followed by erythromycin (81.8%), while no resistance was recorded to phenolics and Quinolones. Four patterns of antimicrobial resistance and four isolates showed multiple antimicrobial resistances to drugs of at least three families. MAR indices were between 0.16 to 0.5; maximum MAR index was assigned to the isolates that exhibited resistance to six antibiotics (Table 3). Four isolates were MDR.

**Table 2:** Antimicrobial resistance profile of *V. cholerae* and *V. parahaemolyticus* isolated from the examined samples.

Antibiotic class	Antibiotic	S	I R	R
Penicillins	Ampicillin	0	0	11 (100%)
	Ampicillin-Sulbactam	0	0	11 (100%)
Cephems	Ceftazidime	7 (63.6%)	0	4 (36.4%)
	Cefotaxime	11 (100%)	0	0
Aminoglycosides	Gentamicin	7 (63.6%)	0	4 (36.4%)
Tetracyclines	Tetracycline	9 (81.8%)	0	2 (18.2%)
Fluoroquinolones	Ciprofloxacin	11 (100%)	0	0
Quinolones	Nalidixic Acid	11 (100%)	0	0
Folate Pathway Inhibitors	Sulfonamides	11 (100)	0	0
Macrolides	Erythromycin	2 (18.2%)	0	9 (81.8%)
Phenolics	Chloramphenicol	9 (81.8%)	2 (18.2%)	0
Carbapenem	Meropenem	11 (100%)	0	0

**Table 3:** Multiple antibiotic resistance (MAR) index of *V. cholerae* and *V. parahaemolyticus* isolates.

Resistance pattern	Frequency of occurrence	*Vibrio* spp. (source)	(No.)	MAR index
AMP + SAM + CAZ + GEN + TE + E	2	*V. cholerae*(raw milk)	1	0.5
		*V. parahaemolyticus* (water)	1	
AMP + SAM + CAZ + GEN + E	2	*V. cholerae*(water)	1	0.4
		*V. parahaemolyticus* (water)	1	
AMP + SAM + E	5	*V. cholerae* (kareish cheese and water)	2	0.25
		*V. parahaemolyticus* (cheese and water)	3	
AMP + SAM	2	*V. cholerae* (raw milk)	1	0.16
		*V. parahaemolyticus* (raw milk)	1	

**Figure 2:**
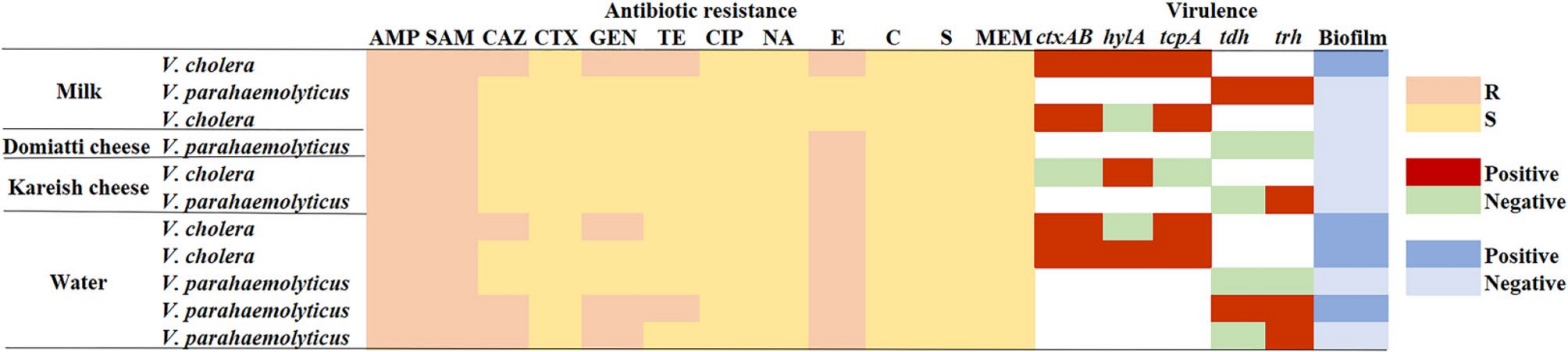
Heat-map summary of antimicrobial resistance, virulence, and biofilm formation by *V. cholera*e and *V. parahaemolyticus* strains isolated from the examined samples.

The virulence genes in the *V. cholerae* and *V. parahaemolyticus* isolates are displayed in Table 4. The biomarker genes, *ctxAB*, *hly*A and *tcp*A, codes for the virulence factors of *V. cholerae* were detected in 80, 60 and 80% of the obtained isolates, respectively. Meanwhile, there are two *trh* + and two *tdh* + /*trh* + *V. parahaemolyticus* strains, accounting for 33.3% per each pattern. Four isolates were moderate biofilm producer and three of them were MDR. All characters of the identified isolated are displayed in Fig. 2.

**Table 4:** Distribution of the virulence-associated genes in *V. cholerae* and *V. parahaemolyticus* isolated from the examined samples.

Samples	Serogroup of *V . Cholerae *(No.)	*V. cholerae* virulence genes (%)			*V. parahaemolyticus* virulence genes (%)			
		*ctxAB*	*hlyA*	*tcpA*	No	*tdh*	*trh*	*tdh*/*trh*
Raw milk	O1 (2)	2(100)	1(50%)	2(100)	1	0	0	1(100)
Domiatti cheese	0	–	–	–	1	0	0	0
Kareish cheese	Non-O1/non O139 (1)	0	1(100)	0	1	0	1(100)	0
Sub-total	3	2(66.7)	2(66.7)	2(66.7)	3	0	1(33.3)	1(33.3)
Water	O1 (2)	2(100)	1(50)	2(100)	3	0	1(33.3)	1(33.3)
Total	5	4 (80)	3 (60)	4 (80)	6	0	2(33.3)	2(33.3)

## Discussion

*Vibrio cholerae* is a genus of zoonotic bacteria with global economic and health significance. It is a significant universal public health burden, producing significant morbidity and mortality in the population. Over the years, a number of researchers have focused on the severity of infections caused by *V. cholerae*, ignoring relatively minor *Vibrio* species that are important medically and some of which are emerging pathogens that can cause mild to severe human diseases^13^**.** A variety of methods were used in this study to isolate and identify the *vibrio* species, including TCBS culturing, the using of *Vibrio* housekeeping *16S rDNA* gene primers, and the amplification and sequencing of the *ctx* and *tox*R genes. Huq et al.^14^ reported that molecular detection approaches had enhanced the incidence of finding harmful microbes while conventional culture-based detection methods might have failed. Many authors endorsed the using of *16S rDNA* gene to identify *Vibrio* spp^15–18^. In addition, the genetic information gained from the sequencing of the *ctx* and *tox*R genes distinguishes between species that are closely related, such as *V. cholerae* and *V. parahaemolyticus*^19^*.* The phylogenetic analysis of the previously sequenced genes revealed a relationship between our strains isolated from milk, milk products, and water and phylogenetically related reference strains on GenBank isolated from water, food, and patients. These findings may provide information about the source of contamination^20–22^. In the current investigation, three of the examined dairy samples included *V. cholerae*, of which two were isolated from raw milk. Tahoun et al.^23^, Sharma et al.^24^, and Islam et al.^25^ also found higher results. This is corroborated by findings from Waturangi et al.^26^ who found that *V. cholerae* could survive for at least three months in all items tested, including UHT milk and ice cream that was kept in a freezer.

Several processes are involved in the production of Domiati cheese, including natural milk fermentation, salting, renneting, and salted whey solutions. The salt in cheese milk, which can range from 5.46 to 9.50%^27^, plays an essential role in the production and processing process by promoting or suppressing bacterial development. *Vibrio* spp. are directly tied to a marine environment and are salt tolerant. According to several investigations *Vibrio* spp. and other bacteria associated with a marine environment were found in the cheese flora^27–30^. The usage of contaminated raw milk and/or contaminated water may have promoted the occurrence of *V. cholerae* and *V. parahaemolyticus* in Kariesh cheese, a popular type of artisanal raw milk soft cheese in Egypt.

*Vibrio* spp. were absent from ice cream and yoghurt samples. Similar finding were reported by Kim et al^31^. Contrarily, Islam et al.^32^ detected *Vibrio* spp. in street ice cream and Tahoun et al.^23^ recovered *V. cholerae* and *V. parahaemolyticus* from yoghurt samples.

*Vibrio cholerae* was also isolated from 2% of the examined freshwater samples. Higher results obtained by Ismail et al.^33^. Although *V. parahaemolyticus*, is a halophilic bacterium and linked to marine water^34,35^, it could be isolated from dairy and freshwater samples. The presence of *V. parahaemolyticus* in dairy samples is supported by the findings of Tahoun et al.^23^ who also recommended that further investigation be performed to ensure its ability to survive in milk. It worth mentioning that the salinity of the Nile elevates from Aswan to Cairo because of lowering the flow of the Nile water^36^, and the elevation continues in the direction of the Mediterranean Sea Nile outlets, where the maximum salinity values were registered^33^ resulting in survival of *V. parahaemolyticus* in certain niches of fresh water and the scenario of subsequent contamination of food through the use of this water in different processing steps is potentially existed. Due to *V. parahaemolyticus* is found in water and the aquatic environment in general^37,38^, it is possible for it to get to milk and its products through utensils washed with contaminated water. Also there are other possible means accelerated the bacterial contamination, including; contaminated hands, unsanitary manufacturing unit conditions, and poor materials quality^39^.

Furthermore, recent discoveries confirming the presence of *V. cholerae* in river sediments demonstrate that the risk of infection associated with river exposure may increase under conditions of sediment resuspension^40^. Isolation of *V. cholerae* from river sediments improved the knowledge of the potential *V. cholerae* sources that accounted for the disease epidemics and have afflicted different developing and Sub-Saharan African countries^13^*.*

*Vibrio cholerae* in raw milk was most likely caused by contamination with farm soil during milking and poor dairy farm management^40^. Workers' unclean hands, poor milk quality, unsanitary circumstances in the manufacturing unit, inferior quality of materials utilised, and water given for washing utensils could all be factors in encouraging bacterial contamination of milk products and post-manufacturing contamination^23,33,39,41–43^.

To assess the actual risk to human health posed by the presence of *V. cholerae* and *V. parahaemolyticus* in the studied samples, identification of the microbe should be followed by PCR detection of the virulence genes responsible for cholera toxin production (CTX) and toxin co-regulated pilus (TCP) in *V. cholerae* and TDH and TRH toxins in *V. parahaemolyticus*. Pathogenic *V. cholerae* strains have two distinct important genetic elements, the cholera toxin element (CTX) and the Vibrio pathogenicity island (VPI), both of which are involved in the coding of the cholera toxin (CTX) and the toxin co-regulated pilus (TCP), respectively^44^**.** Only the O1 and O139 serogroups of *V. cholerae* are known to cause epidemic or pandemic cholera. Serogroup O1 has been linked to pandemics since 1899, and serogroup O139 has been linked to pandemics since 1992, with both serogroups being found in recent outbreaks of cholera^45,46^. In serotyping of pathogenic isolates (O1 and O139), combined genotypic and phenotypic analyses have largely replaced serotyping^47^*.*

*V. cholerae* O1 isolates obtained from raw milk samples harbored *ctx*AB and *tcp*A genes, indicating that the genes encoding the virulence and surface organelles needed for intestinal attachment and colonisation were preserved. As a result, both strains maintained the core of the CTX genetic element as well as TCP, both of which are regarded as essential characteristics in pathogenicity. Similar results reported by Tahoun et al.^23^ where all *V. cholerae* isolates from milk belonged to O1 serogroup and also, they were positive for the *ctxAB*, *hly*A, and *tcp*A genes. The virulent genes *ctxA* and *toxR* were not found in any of the isolates studied by Meena et al.^48^, but the *hly*A gene was identified as the dominant biomarker gene among the isolates.

The non-O1/O139 *V. cholerae* strains rarely possess cholera toxin, which is encoded by the *ctx*AB gene, but they can cause less severe diarrhea-like symptoms^49^ and the enterotoxic activity is most likely due to the produced hemolysin^50^**.** Out of the five *V. cholerae* isolates, only one strain was non-O1/O139 and carried only the virulence *hly*A gene. Contradictory findings were obtained by Ahmed et al.^43^, Sarkar et al.^44^, Bakhshi et al.^51^. Serogroup non O1/O139 have been associated to a large number of cases of diarrhoea in different regions of the world, including Sudan in 1968^44,52,53^. The lack of the *ctx* gene in this serogroup of *V. cholerae* does not preclude the risk posed by its existence^54,55^. Previous studies have shown that antigenic translation of *V. cholerae* non O1/O139 to *V. cholerae* O1 takes place under favourable conditions^56–58^.

DH-related hemolysin (*trh*) and thermostable direct hemolysin (*tdh*) of *V. parahaemolyticus* have comparable hemolytic activity on living cells and induce erythrocyte lysis^59^. About 0.2–3% of environmental *V. parahaemolyticus* isolates are potentially pathogenic owing to the occurrence of *tdh* and/or *trh* genes, as per studies carried out in various areas^60^. Of the six examined *V. parahaemolyticus* isolates from dairy and water samples, 33.3% of each were positive for both *tdh* and/or *trh* genes. Similar results reported by Tahoun et al.^23^ Also, Ahmed et al.^43^ found that 33.3% of the obtained isolates harboured both *tdh* and *trh* genes and Abd-Elghany and Sallam^61^ detected *tdh* and/or *trh* genes in 11.1% of the isolates. These findings are supported by the fact that not all of *V. parahaemolyticus* strains are pathogenic, and the pathogenic ones that are associated with the majority of illness and deaths are distinguished by producing TDH and/or TRH hemolysin encoded by *tdh* and *trh* genes, respectively^62^.The majority of *V. parahaemolyticus* strains of environmental or from food origin are not pathogenic to humans^63^. Two out of six isolate harboured *trh* gene only. TDH negative strains were found to produce TRH and are also considered pathogenic to man^64,65^.

The presence of this bacterium species in the aquatic environment raises human worries about food safety since, depending on environmental conditions, it has the ability to produce disease outbreaks^66^. The Centre for Disease and Control (CDC) recommends using antibiotics combined with fluid replacement to treat *Vibrio* infections^67^. Most antibiotics tested, including ampicillin, chloramphenicol, ciprofloxacin, gentamicin, erythromycin, quinolone and tetracycline are prescribed as first-line antibiotics for *Vibrio* infections treatmen^8,67–69^. The emergence of antibiotic resistance is a challenging problem that repetitively links human, environmental, and pathogen-related characteristics^70^.

The ampicillin and ampicillin-sulbactam resistance by of all isolates noted in the current study is consistent with the CLSI standards^71^, that notified *Vibrio spp.* as inherently resistant to ampicillin, which also is in line with previous studies^23,24,43,48^. The development of resistant bacteria to the penicillin antibiotic class in past years has restricted its effectiveness as one of the most effective antibiotics in primary care^72^. The isolates in this study were extremely resistant to erythromycin, ceftazidime, and Gentamicin. This was supported by Sharma and Malik^24^, Islam^25^. and Ahmed et al.^43^ who also recorded high resistance to Ciprofloxacin, Nalidixic acid and Cefotaxime in contrast to this study. Unlike this study, all *Vibrio* isolates obtained by Meena et al.^48^ were sensitive to erythromycin but on the other hand similar results are recorded for Ciprofloxacin and Nalidixic acid drugs by them.

MAR indices between 0.16 to 0.5; maximum MAR index was contributed to the isolates displayed resistance to six antibiotics and four out of 11 *Vibrio* isolates were MDR with. This is consistent with Tahoun et al.^23^ who found that most isolates showed MDR with MAR index ranging from 0.15 to 0.54, and Islam et al.^25^ and Ahmed et al.^43^ who found that all the isolates showed MDR with an MAR index ranging from 0.58 to 1. Meena et al.^48^ recorded MAR index ranging from 0.11 to 0.22. Elevated levels of MDR may be a result of the increased opportunity for resistance genes located on plasmids to be exchanged among environmental isolates via horizontal gene transfer due to the widespread, unsupervised use of antimicrobials in the infection treatment^73,74^.

A MAR higher than 0.2 indicates that high-risk sources, like farmers and farm animals that regularly take antibiotics, are the source of contamination, posing a threat to consumers. The current study found high MAR indices in water isolates, suggesting that these isolates came from high-risk sources; thus, antimicrobial resistance monitoring is critical for ascertaining the efficacy of new antibiotics and ensuring food safety^75^. Municipal and industrial waste water have been outlined as a potential source of resistant isolates in the aquatic ecosystem. A significant amount of the antibiotics that people take for medical reasons are expelled in their faeces and urine in an active biological form^76–78^and between 30 to 90% of the antibiotics that animals consume are also eliminated in faeces and urine^79^. Antibiotic-resistant bacteria and antibiotics were found to pollute the environment through animal excreta^80,81^. This phenomenon was newly confirmed in a study of 20 calf farms in the Netherlands. Antibiotics were found in 75% and 95% of the calf faeces and cattle farms, respectively, and the most common residual antibiotics recovered were oxytetracycline, doxycycline, and sulfadiazine^78^. It is a possible scenario for water contamination and subsequent contamination of milk and dairy products with antibiotic resistant pathogens.

The US National Institutes of Health reported that biofilm is accountable for more than 80% of bacterial diseases^82^. Extracellular polymeric substances produced by biofilm-producing bacteria, such as *Vibrio*, provide an appropriate medium for surface colonialization to produce biofilms. Once the biofilm is formed, the structure of the biofilm permits bacteria to remain alive and thrive in adverse environments such as high salinity and antibiotics^83^. Four isolates are biofilm producers, three recovered from water samples and one from raw milk samples and most of them are *V. cholerae*. *Vibrio* spp. ability to produce biofilm were recorded by various studies^6,84,85^. Three isolates are MDR this is supported by the finding that biofilm is related to appearance of multidrug resistance^86^.

*V. cholerae*'s ability to form biofilms is essential to the colonisation of the intestine; however, the biofilm structure generated throughout infection, in addition to their involvement in intestinal colonisation and pathogenicity, currently unclear^2^. It has been reported that *V. cholerae* biofilms are more resistant to acid inactivation^87^. Furthermore, biofilm-derived cells have competitive advantage over planktonic cells when it comes to limited nutrients in the small intestine^88^. Planktonic cells that disengaged from an infecting biofilm should move toward the intestinal mucosa, where they must break through the mucus barrier and proceed to the underlying epithelium. *Vibrio* cells that fail to form biofilm-like aggregates within the mucus gel due to lack of encoding genes (*vps*) or their expression^89^and/or penetrate the protective mucosa are leached inertly as a consequence of persistent mucosal degeneration^90^.

Given that getting cholera includes the oral intake of virulent *Vibrio* cells that can express TCP and CT in the pattern of planktonic cells or biofilms and infection is contracted naturally in the latter form which represents a fast pathway for disease spreading during outbreaks^2^, also *V. cholerae* cells in a biofilm demonstrate a smaller infective dose and totally dominate their planktonic cells’ colonisation^91^, and biofilms mostly require greater concentrations of antibiotics to be eliminated than planktonic bacteria^92^, as a consequence, there is a potential that the obtained isolates represent a high potential threat.

In this study, a link was revealed between the patterns of drug resistance and virulence phenotype. Two out of four MDR isolates harboured virulence genes and exhibited phenotypic biofilm capacity. The earlier finding was corroborated by Katongole et al.^93^ who discovered that biofilm-forming organisms harboured more virulence genes and were more MDR than non-biofilm generating pathogens. This is because biofilms are crucial because they act as hubs for horizontal gene transfer, which promotes the spread of virulence and antibiotic resistance genes^94^. These findings raise significant concerns regarding food safety and public health since raw milk may be a reservoir for bacteria that are resistant to antibiotics and virulent strains that may spread to humans through the food supply, and so this concern becomes more severe with regard to high temperatures in the study area, this is in line with reports of *V. cholerae* outbreaks occurring in a stressful environment of high temperatures during the spring and summer seasons^95,96^. Furthermore, the result presented here are is consistent with the findings of many authors who have shown that the introduction of *V. cholerae* O1 into nonendemic areas of less-than-ideal sanitation frequently causes the disease to spread more quickly. This is done by means of a fast faecal-oral pathway that takes advantage of the transitory hyper infective stage of *V. cholerae* found in fresh cholera stool^97–100^.

## Conclusions

The use of TCBS agar and PCR in the isolation and identification of *Vibrio* spp. is critical for biotype detection and differentiation. The isolation of *V. cholerae* and *V. parahaemolyticus* from dairy and water samples necessitates application of strict hygienic measures. Most *Vibrio* isolates were virulent, exhibited MDR with a high MAR index and four isolates were biofilm producer, representing public health hazard.

## Materials and methods

### Sample collection

The present study was designed to determine the prevalence of *Vibrio* spp. in 300 raw milk and dairy products were collected from farmers’ houses, dairy farms, local dairy shops and vendors in Qena, Egypt. These samples included 100 raw cow and buffalo milk (50 samples each), and cheese, yoghurt and ice cream (50 samples each). Additionally, 100 water samples were collected from farmer's houses and dairy farms where the raw milk was collected. These samples were collected from May to October 2021.

### Isolation and identification of *Vibrio* species

The Food and Drug Administration's Bacteriological Analytical Manual (FDA 2004)^101^ was followed for the isolation of *Vibrio* species. Briefly, one ml/g of each milk and water samples was blended with 9 ml of sterile alkaline peptone water (APW) (Oxoid, CM1028, UK) while 10 g for each Yoghurt, cheese, ice-cream were blended in 90 ml APW and incubated at 35 °C ± 2 °C for 24–48 h (ISO-TS-21872–1, 2007)^102^. A loopful of the enriched broth, was streaked onto Cholera Medium TCBS agar (Oxoid, CM0333, UK), then plates were incubated at 37 °C for 24 h. Presumptive *Vibrio* colonies (yellow, blue green) were streaked on tryptone soya agar (Oxoid, CM0131, UK) containing 2% NaCl for purification. Gram staining and biochemical tests including; growth in 0,1% NaCl, oxidase, catalase, motility, glucose oxidation/fermentation, gelatinase production, nitrate reduction, arginine dehydrolase utilization, lysine and ornithine decarboxylase utilization, mannose, arabinose, sucrose and lactose sucrose mannose were used for identification^103^.

### Molecular characterization of *Vibrio* spp. strains

Uniplex and multiplex PCR reactions used in molecular identification of *Vibrio* spp. The primers and target genes used in this study, as well as the PCR cycling conditions and amplicon sizes, are described in their respective references and are listed in Supplementary Table 1. PCR amplification was carried out in an Applied Biosystem 2720 thermal cycler. To validate the presence of amplified DNA, PCR products were examined using 1.5% (w/v) agarose gel electrophoreses in 0.5 TBE buffer at a constant voltage of 90 V. A gel documentation system (Alpha Innotech, Biometra, Göttingen, Germany) used to photograph the gel.

#### Preparation of genomic DNA

Genomic DNA was extracted with the DNA extraction QIAamp DNA Mini kit (Qiagen GmbH, Hilden, Germany). DNA was extracted from 1 ml of overnight-grown cultures in tryptic soy broth medium (TSB) (Oxoid, CM0129, UK) and reconstituted in 100 μl of DNA hydration buffer.

#### Molecular identification of the obtained isolates

Confirmation of the biochemically identified *Vibrio* colonies was done by PCR using housekeeping *16S rDNA* gene primers set for *Vibrio* species^104^. Specific primers targeting the *toxR* and *ctx* genes were used to confirm *V. parahaemolyticus* and *V. cholerae*^105,106^. Serotyping of *V. cholerae* isolates was molecularly performed using O1-rfb and O139-rfb genes^107^.

Sequencing of the *ctx* and *toxR* genes was done to confirm the molecular identification of *V. cholerae* and *V. parahaemolyticus* isolates. PCR products were purified using a QIAquick PCR Purification Kit as directed by the manufacturer (Qiagen, Valencia, CA). The sequence reaction was conducted with a Bigdye Terminator V3.1 cycle sequencing kit (Thermo Fisher Scientific in the United States), and it was purified with a Centrisep spin column. The genetic analyzer Applied Biosystems 3130 (HITACHI, Tokyo, Japan) was used to obtain DNA sequences. All the obtained sequences of *ctx and tox*R genes were analysed using Lasergene (version 7.2; DNASTAR, Madison, WI) and submitted to the GenBank (https://blast.ncbi.nlm.nih.gov/Blast.cgi?PROGRAM=blastn&PAGE_TYPE=BlastSearch&LINK_LOC=blasthome). The strains of *V. cholerae* MN829555.1, CP042299.1, CP047059.1, CP046837.1, CP047295.1, CP046840.1, CP046840.1, CP047299.1, CP047301.1 and CP046745.1 and *V. parahaemolyticus* CP028342.1, CP028141.1, CP078627.1, DQ845167.1, DQ845167.1, CP041202.1, MF983554.1, AB372520.1, CP023485.1, CP031781.1, MF983555.1, CP064035.1, CP078735.1, CP068627.1 and AB300874.1 from the NCBI database were included in the phylogenetic analysis. The sequence alignment and phylogenetic tree were carried out using multiple alignment algorithms in MegAlign (version 10.2.4; DNASTAR, Wisconsin, USA).

#### Molecular detection of virulence determinants

*V. cholerae* isolates were characterized for their virulence using specific primers targeting the *ctx*AB^49^ the *hly*A^108^ and the *tcp*A^108^virulence determinants. While *V. parahaemolyticus* isolates were tested for *tdh* and *trh* virulence genes^109^.

### Antimicrobial susceptibility test

Antimicrobial resistance of *V. cholerae* and *V. parahaemolyticus* isolates (n = 11) was determined using disk diffusion method on Mueller Hinton Agar (Oxoid, UK) according to the guidelines by the Clinical and Laboratory Standards Institute (CLSI,2015)^110^. Twelve antibiotic disks (Oxoid, UK) used in this study including ampicillin (AMP) (20 μg), Ampicillin-sulbactam (SAM) (10 μg), Ceftazidime (CAZ) (30 μg), Cefotaxime (CTX) (30 μg), Tetracycline (TE) (30 μg), Ciprofloxacin (CIP) (5 μg), Gentamicin (GEN) (10 μg), Nalidixic Acid (NA) (30 μg), Erythromycin (E) (15 μg), Sulfonamides (S) (300 μg), Chloramphenicol (C) (30 μg), and Meropenem (MEM) (10 μg). The zones of inhibition were measured and compared to the world standards CLSI, isolates were recorded as Resistant (R), and Sensitive (S) accordingly. For nalidixic acid (NA), the *Enterobacteriaceae* interpretation criteria was used.

#### Determination of MAR index

The method described by Osundiya et al.^111^ was used to generate the MAR index^107^, in which the number of antibiotics to which an isolate is resistant (a) is divided by the total number of antibiotics used in the study (b). The formula for calculating is illustrated below:

$${\text{MAR}}\;{\text{Index = a }}/{\text{ b}}.$$

### Biofilm formation

The biofilm production of bacterial strains was investigated using a microtiter plate assay as per^112^. Briefly, bacteria were cultured for 24 h in Tryptone Soy Broth (TSB) (Oxoid, CM0129B, UK) supplemented with 3% NaCl at 37 °C. In fresh TSB (3% NaCl), 200 µL of a 1:100 dilution of overnight cultures was adjusted to 0.5 McFarland turbidity. The diluted solutions were then distributed into the wells, and the plats were incubated at 37 °C for 24 h. Three well of uninoculated TSB with 3% NaCl were set as the control. The wells' contents were discarded, and the wells were rinsed twice with phosphate buffer saline (PBS). Each well received 200 µL of crystal violet dye (1%) before being incubated at room temperature (25 °C) for 1 h. The staining dye was removed, and the wells were rinsed three times with PBS before being allowed to air-dry at 25 °C. Then 200 µL of acetic acid (33%) for 30 min were added to each well to resolubilize the adherent cells and the optical density at 570 nm was measured. Three separate tests were carried out. All isolates' biofilm-producing ability was classified according Stepanović et al.^113^ as follows: No biofilm formation if OD test < OD control; weak biofilm formation if OD control < OD test < 2OD control; moderate biofilm formation if 2ODcontrol < OD test < 4OD control; and strong biofilm formation if OD test > 4OD.

### Statistical analysis

The mean and standard deviation of the data were displayed. The Graph Prism 8 one-way ANOVA test was employed. The criterion for significance was *P* < 0.05.

### Ethical approval

The study was approved by the Animal Ethics Committee for Veterinary Research (75/02. 10.2022), Faculty of Veterinary Medicine, South Valley University, Qena, Egypt.

### Supplementary Information

Supplementary Table.

## Data Availability

Dr. Mon A. El-Zamkan and Hams M. A. Mohamed have data available upon request.

## References

[1] Ghosh AK, Panda SK, Luyten W (2021). Anti-vibrio and immune-enhancing activity of medicinal plants in shrimp: A comprehensive review. Fish Shellfish Immunol..

[2] Silva AJ, Benitez JA (2016). Vibrio cholerae biofilms and cholera pathogenesis. PLoS Negl. Trop. Dis..

[3] Robert-Pillot A, Copin S, Himber C, Gay M, Quilici M-L (2014). Occurrence of the three major Vibrio species pathogenic for human in seafood products consumed in France using real-time PCR. Int. J. Food Microbiol..

[4] Su Y-C, Liu C (2007). Vibrio parahaemolyticus: A concern of seafood safety. Food Microbiol..

[5] Broberg CA, Calder TJ, Orth K (2011). Vibrio parahaemolyticus cell biology and pathogenicity determinants. Microbes Infect..

[6] Wang D (2022). Global expansion of Vibrio parahaemolyticus threatens the seafood industry: Perspective on controlling its biofilm formation. LWT.

[7] Wang Y, Zhao Y, Pan Y, Liu H (2020). Comparison on the growth variability of Vibrio parahaemolyticus coupled with strain sources and genotypes analyses in simulated gastric digestion fluids. Front. Microbiol..

[8] Laganà P, Caruso G, Minutoli E, Zaccone R, Delia S (2011). Susceptibility to antibiotics of Vibrio spp. and Photobacterium damsela ssp. piscicida strains isolated from Italian aquaculture farms. New Microbiol..

[9] Yano Y (2014). Prevalence and antimicrobial susceptibility of Vibrio species related to food safety isolated from shrimp cultured at inland ponds in Thailand. Food Control.

[10] Tendencia EA, de la Peña LD (2001). Antibiotic resistance of bacteria from shrimp ponds. Aquaculture.

[11] Duran GM, Marshall DL (2005). Ready-to-eat shrimp as an international vehicle of antibiotic-resistant bacteria. J. Food Prot..

[12] Guglielmetti E, Korhonen JM, Heikkinen J, Morelli L, Von Wright A (2009). Transfer of plasmid-mediated resistance to tetracycline in pathogenic bacteria from fish and aquaculture environments. FEMS Microbiol. Lett..

[13] Osunla CA, Okoh AI (2017). Vibrio pathogens: A public health concern in rural water resources in sub-Saharan Africa. Int. J. Environ. Res. Public Health.

[14] Huq A (2012). Detection, isolation, and identification of Vibrio cholerae from the environment. Curr. Protoc. Microbiol..

[15] Al-Saady AT, Baqer KA, Al-Salim ZKS (2020). Molecular detection and phylogenetic analysis of Vibrio cholerae genotypes in Hillah Iraq. New Microbes New Infect..

[16] Hasan MAR, Siddique MA, Hasan M, Hossain MA, Rahman MS (2017). 16S rRNA gene sequence based identification of Vibrio spp. in shrimp and tilapia hatcheries of Bangladesh. Dhaka Univ. J. Biol. Sci..

[17] Azwai SM (2016). Isolation and molecular identification of Vibrio spp. by sequencing of 16S rDNA from seafood, meat and meat products in Libya. Open Vet. J..

[18] Church DL (2020). Performance and application of 16S rRNA gene cycle sequencing for routine identification of bacteria in the clinical microbiology laboratory. Clin. Microbiol. Rev..

[19] Baker-Austin C (2018). Vibrio spp. infections. Nat. Rev. Dis. Prim..

[20] Naha A (2020). Deciphering the possible role of ctxB7 allele on higher production of cholera toxin by Haitian variant Vibrio cholerae O1. PLoS Negl. Trop. Dis..

[21] Vongxay K (2008). Occurrence of pandemic clones of Vibrio parahaemolyticus isolates from seafood and clinical samples in a Chinese coastal province. Foodborne Pathog. Dis..

[22] Miyahara M, Arakawa E (2010). Seasonal changes in the detection of Vibrio parahaemolyticus in bivalves purchased from a retail store. Bokin Bobai-J. Antibact. Antifung. Agents.

[23] Tahoun ABMB (2021). Genotypic characterization and antimicrobial resistance of Vibrio cholerae and Vibrio parahaemolyticus isolated from milk, dairy products, and humans with respect to inhibitory activity of a probiotic Lactobacillus rhamenosus. LWT.

[24] Sharma D, Malik A (2012). Incidence and prevalence of antimicrobial resistant Vibrio cholerae from dairy farms. African J. Microbiol. Res..

[25] Islam KI, Kabir SL, Saha S, Khan M (2013). Prevalence and antimicrobial resistance patterns of Vibrio Cholerae from Bangladesh Agricultural University dairy farm. Int. J. Med. Sci. Biotechnol..

[26] Waturangi DE, Amadeus S, Kelvianto YE (2015). Survival of enteroaggregative Escherichia coli and Vibrio cholerae in frozen and chilled foods. J. Infect. Dev. Ctries..

[27] Mounier J (2005). Surface microflora of four smear-ripened cheeses. Appl. Environ. Microbiol..

[28] Møretrø T, Langsrud S (2017). Residential bacteria on surfaces in the food industry and their implications for food safety and quality. Compr. Rev. Food Sci. Food Saf..

[29] O’Sullivan DJ (2015). Temporal and spatial differences in microbial composition during the manufacture of a continental-type cheese. Appl. Environ. Microbiol..

[30] El-Baradei G, Delacroix-Buchet A, Ogier J-C (2007). Biodiversity of bacterial ecosystems in traditional Egyptian Domiati cheese. Appl. Environ. Microbiol..

[31] Kim MJ, Kim SA, Kang YS, Hwang IG, Rhee MS (2013). Microbial diversity and prevalence of foodborne pathogens in cheap and junk foods consumed by primary schoolchildren. Lett. Appl. Microbiol..

[32] Islam MT, Amin MR, Hoque SMR, Alim SR (2014). Microbial loads and association of enteropathogenic bacteria in ice-creams sold by street vendors at Dhaka city in Bangladesh. Int. J. Pharm. Sci. Res..

[33] Ismail EM (2021). Ecoepidemiology and potential transmission of Vibrio cholerae among different environmental niches: An upcoming threat in Egypt. Pathogens.

[34] Letchumanan V, Chan K-G, Lee L-H (2014). Vibrio parahaemolyticus: A review on the pathogenesis, prevalence, and advance molecular identification techniques. Front. Microbiol..

[35] Almejhim M, Aljeldah M, Elhadi N (2021). Improved isolation and detection of toxigenic Vibrio parahaemolyticus from coastal water in Saudi Arabia using immunomagnetic enrichment. PeerJ.

[36] El-Agha DE, Closas A, Molle F (2017). Below the radar: The boom of groundwater use in the central part of the Nile Delta in Egypt. Hydrogeol. J..

[37] Caburlotto G (2016). Occurrence and molecular characterisation of Vibrio parahaemolyticus in crustaceans commercialised in Venice area, Italy. Int. J. Food Microbiol..

[38] Xie T, Wu Q, Xu X, Zhang J, Guo W (2015). Prevalence and population analysis of Vibrio parahaemolyticus in aquatic products from South China markets. FEMS Microbiol. Lett..

[39] Kulshrestha SB (1990). Prevalence of enteropathogenic serogroups of E coli in milk products samples from Bareilly and their multiple drug resistance. Indian J. Dairy Sci..

[40] Abia ALK, Ubomba-Jaswa E, Genthe B, Momba MNB (2016). Quantitative microbial risk assessment (QMRA) shows increased public health risk associated with exposure to river water under conditions of riverbed sediment resuspension. Sci. Total Environ..

[41] Maheshwari M, Krishnaiah N, Ramana D (2011). Evaluation of polymerase chain reaction for the detection of vibrio cholerae in contaminants. Ann. Biol. Res..

[42] Grewal JS, Tiwari RP (1990). Microbiological quality of rasmalai. J. Food Sci. Technol..

[43] Ahmed HA (2018). Molecular characterization, antibiotic resistance pattern and biofilm formation of Vibrio parahaemolyticus and V cholerae isolated from crustaceans and humans. Int. J. Food Microbiol..

[44] Sarker A (2002). Vibrio pathogenicity island and cholera toxin genetic element-associated virulence genes and their ecpression in non-O1 non-O139 strains of Vibrio cholerae. Infect. Immun..

[45] Le Roux, W. J., Schaefer, L. M., Venter, S. N. Vibrio cholerae and cholera: A recent African perspective. *Curr. Microbiol. Res. Africa Sel. Appl. Sustain. Environ. Manag.* 69–113 (2020).

[46] Cravioto, A., Lanata, C. F., Lantagne, D. S. & Balakrish Nair, G. Final report of the independent panel of experts on the cholera outbreak in Haiti. in *Final Report of the Independent Panel of Experts on the Cholera Outbreak in Haiti* 32 (2011).

[47] Hasan NA (2012). Genomic diversity of 2010 Haitian cholera outbreak strains. Proc. Natl. Acad. Sci..

[48] Meena B (2019). Studies on diversity of Vibrio sp and the prevalence of hapA, tcpI, st, rtxA&C, acfB, hlyA, ctxA, ompU and toxR genes in environmental strains of Vibrio cholerae from Port Blair bays of South Andaman. India. Mar. Pollut. Bull..

[49] de Menezes FGR (2014). Detection of virulence genes in environmental strains of Vibrio cholerae from estuaries in northeastern Brazil. Rev. Inst. Med. Trop. Sao Paulo.

[50] Rivera ING, Chun J, Huq A, Sack RB, Colwell RR (2001). Genotypes associated with virulence in environmental isolates of Vibrio cholerae. Appl. Environ. Microbiol..

[51] Bakhshi B (2012). Presence of CTX gene cluster in environmental non-O1/O139 Vibrio cholerae and its potential clinical significance. Indian J. Med. Microbiol..

[52] Dorman MJ, Thomson NR (2023). Vibrio cholerae O37: one of the exceptions that prove the rule. Microb. Genomics.

[53] Dorman MJ (2019). High quality reference genomes for toxigenic and non-toxigenic Vibrio cholerae serogroup O139. Sci. Rep..

[54] Marin MA (2013). Cholera outbreaks in Nigeria are associated with multidrug resistant atypical El Tor and non-O1/non-O139 Vibrio cholerae. PLoS Negl. Trop. Dis..

[55] Engel MF, Muijsken MA, Mooi-Kokenberg E, Kuijper EJ, van Westerloo DJ (2016). Vibrio cholerae non-O1 bacteraemia: description of three cases in the Netherlands and a literature review. Eurosurveillance.

[56] Montilla R, Chowdhury MAR, Huq A, Xu B, Colwell RR (1996). Serogroup conversion of Vibrio cholerae non-O1 to Vibrio cholerae O1: Effect of growth state of cells, temperature, and salinity. Can. J. Microbiol..

[57] Blokesch M, Schoolnik GK (2007). Serogroup conversion of Vibrio cholerae in aquatic reservoirs. PLoS Pathog..

[58] Li M, Shimada T, Morris JG, Sulakvelidze A, Sozhamannan S (2003). Evidence for the emergence of non-O1 and non-O139 Vibrio cholerae strains with pathogenic potential by exchange of O-antigen biosynthesis regions. Infect. Immun..

[59] Makino K (2003). Genome sequence of Vibrio parahaemolyticus: a pathogenic mechanism distinct from that of V cholerae. Lancet.

[60] Nordstrom JL, Vickery MCL, Blackstone GM, Murray SL, DePaola A (2007). Development of a multiplex real-time PCR assay with an internal amplification control for the detection of total and pathogenic Vibrio parahaemolyticus bacteria in oysters. Appl. Environ. Microbiol..

[61] Abd-Elghany SM, Sallam KI (2013). Occurrence and molecular identification of Vibrio parahaemolyticus in retail shellfish in Mansoura. Egypt. Food Control.

[62] Villicaña C (2019). Occurrence and abundance of pathogenic Vibrio species in raw oysters at retail seafood markets in northwestern Mexico. J. Food Prot..

[63] Lopatek M, Wieczorek K, Osek J (2018). Antimicrobial resistance, virulence factors, and genetic profiles of Vibrio parahaemolyticus from seafood. Appl. Environ. Microbiol..

[64] Honda T, Ni YX, Miwatani T (1988). Purification and characterization of a hemolysin produced by a clinical isolate of Kanagawa phenomenon-negative Vibrio parahaemolyticus and related to the thermostable direct hemolysin. Infect. Immun..

[65] Li J (2016). Vibrio parahaemolyticus strains of pandemic serotypes identified from clinical and environmental samples from Jiangsu China. Front. Microbiol..

[66] Okocha RC, Olatoye IO, Adedeji OB (2018). Food safety impacts of antimicrobial use and their residues in aquaculture. Public Health Rev..

[67] Prevention, C. for D. C. and. CDC. Antibiotic resistance threats in the United States (2019).

[68] Han F, Walker RD, Janes ME, Prinyawiwatkul W, Ge B (2007). Antimicrobial susceptibilities of Vibrio parahaemolyticus and Vibrio vulnificus isolates from Louisiana Gulf and retail raw oysters. Appl. Environ. Microbiol..

[69] Kitaoka M, Miyata ST, Unterweger D, Pukatzki S (2011). Antibiotic resistance mechanisms of Vibrio cholerae. J. Med. Microbiol..

[70] Aminov RI (2011). Horizontal gene exchange in environmental microbiota. Front. Microbiol..

[71] CLSI. Title Method for antifungal disk diffusion susceptibility testing of non-dermatophyte filamentous fungi; Approved Guideline, CLSI document M51-A. Wayne, PA: Clinical and Laboratory Standards Institute; (2010).

[72] Miller K, O’Neill AJ, Chopra I (2002). Response of Escherichia coli hypermutators to selection pressure with antimicrobial agents from different classes. J. Antimicrob. Chemother..

[73] Ottaviani D (2013). Extensive investigation of antimicrobial resistance in Vibrio parahaemolyticus from shellfish and clinical sources, Italy. Int. J. Antimicrob. Agents.

[74] Kümmerer K (2009). Antibiotics in the aquatic environment–a review–part II. Chemosphere.

[75] Yu Q (2016). Prevalence and antimicrobial susceptibility of Vibrio parahaemolyticus isolated from retail shellfish in Shanghai. Food Control.

[76] Singer AC (2011). Assessing the ecotoxicologic hazards of a pandemic influenza medical response. Environ. Health Perspect..

[77] Zhang Q-Q, Ying G-G, Pan C-G, Liu Y-S, Zhao J-L (2015). Comprehensive evaluation of antibiotics emission and fate in the river basins of China: source analysis, multimedia modeling, and linkage to bacterial resistance. Environ. Sci. Technol..

[78] Verlicchi P, Zambello E (2016). Predicted and measured concentrations of pharmaceuticals in hospital effluents. Examination of the strengths and weaknesses of the two approaches through the analysis of a case study. Sci. Total Environ..

[79] Berendsen BJA, Wegh RS, Memelink J, Zuidema T, Stolker LAM (2015). The analysis of animal faeces as a tool to monitor antibiotic usage. Talanta.

[80] Udikovic-Kolic N, Wichmann F, Broderick NA, Handelsman J (2014). Bloom of resident antibiotic-resistant bacteria in soil following manure fertilization. Proc. Natl. Acad. Sci..

[81] Wichmann F, Udikovic-Kolic N, Andrew S, Handelsman J (2014). Diverse antibiotic resistance genes in dairy cow manure. MBio.

[82] Hall MR, McGillicuddy E, Kaplan LJ (2014). Biofilm: Basic principles, pathophysiology, and implications for clinicians. Surg. Infect. (Larchmt).

[83] Suhartono, S., Ismail, Y. S., Muhayya, S. R. & Husnah, M. Ethanolic extracts of Moringa oleifera leaves inhibit biofilm formation of Vibrio alginolyticus in vitro. in *IOP Conference Series: Earth and Environmental Science* vol. 348 12018 (IOP Publishing, 2019).

[84] Yan J, Sharo AG, Stone HA, Wingreen NS, Bassler BL (2016). Vibrio cholerae biofilm growth program and architecture revealed by single-cell live imaging. Proc. Natl. Acad. Sci..

[85] Santajit S (2022). Occurrence, antimicrobial resistance, virulence, and biofilm formation capacity of Vibrio spp. and Aeromonas spp. isolated from raw seafood marketed in Bangkok Thailand. Vet. World.

[86] El-Zamkan MA, Mohamed HMA (2021). Antimicrobial resistance, virulence genes and biofilm formation in Enterococcus species isolated from milk of sheep and goat with subclinical mastitis. PLoS ONE.

[87] Dua, P., Karmakar, A. & Ghosh, C. Virulence gene profiles, biofilm formation, and antimicrobial resistance of Vibrio cholerae non-O1/non-O139 bacteria isolated from West Bengal, India. *Heliyon* **4**, (2018). 10.1016/j.heliyon.2018.e01040 PMC6299121 30582054

[88] Mudrak B, Tamayo R (2012). The Vibrio cholerae Pst2 phosphate transport system is upregulated in biofilms and contributes to biofilm-induced hyperinfectivity. Infect. Immun..

[89] Liu Z (2015). Vibrio cholerae represses polysaccharide synthesis to promote motility in mucosa. Infect. Immun..

[90] Millet YA (2014). Insights into Vibrio cholerae intestinal colonization from monitoring fluorescently labeled bacteria. PLoS Pathog..

[91] Tamayo R, Patimalla B, Camilli A (2010). Growth in a biofilm induces a hyperinfectious phenotype in Vibrio cholerae. Infect. Immun..

[92] Olson ME, Ceri H, Morck DW, Buret AG, Read RR (2002). Biofilm bacteria: formation and comparative susceptibility to antibiotics. Can. J. Vet. Res..

[93] Katongole P, Nalubega F, Florence NC, Asiimwe B, Andia I (2020). Biofilm formation, antimicrobial susceptibility and virulence genes of Uropathogenic Escherichia coli isolated from clinical isolates in Uganda. BMC Infect. Dis..

[94] Michaelis C, Grohmann E (2023). Horizontal gene transfer of antibiotic resistance genes in biofilms. Antibiotics.

[95] Rita R (2009). Cholera and climate: A demonstrated relationship. Trans. Am. Clin. Climatol. Assoc..

[96] Jutla AS, Akanda AS, Griffiths JK, Colwell R, Islam S (2011). Warming oceans, phytoplankton, and river discharge: Implications for cholera outbreaks. Am. J. Trop. Med. Hyg..

[97] Butler SM (2006). Cholera stool bacteria repress chemotaxis to increase infectivity. Mol. Microbiol..

[98] Okoh, A. Cholera monitoring and response guidelines. *Water Res. Commun.* (2018).

[99] Gancz, H., & Merrell, D. S. Acid survival mechanisms of bacterial pathogens of the digestive tract. in *Stress response in pathogenic bacteria* 135–166 (CABI Wallingford UK, 2011).

[100] Morris JG (2011). Cholera—modern pandemic disease of ancient lineage. Emerg. Infect. Dis..

[101] 2004, F. No TitleBacteriological analytical manual food and drug administration. [chapter 9].

[102] Scherm B, Palomba M, Serra D, Marcello A, Migheli Q (2005). Detection of transcripts of the aflatoxin genes aflD, aflO, and aflP by reverse transcription–polymerase chain reaction allows differentiation of aflatoxin-producing and non-producing isolates of Aspergillus flavus and Aspergillus parasiticus. Int. J. Food Microbiol..

[103] Farmer JJ, Genus I (2005). Vibrio Pacini 1854, 411^. Bergey’s Man. Syst. Bacteriol..

[104] Tarr CL (2007). Identification of vibrio isolates by a multiplex PCR assay and rpoB sequence determination. J. Clin. Microbiol..

[105] Kim YB (1999). Identification of vibrio parahaemolyticus strains at the species level by PCR targeted to the toxR gene. J. Clin. Microbiol..

[106] Mousavi, S. L., RASOULI, I., Nazarian, S. H. & Amani, J. Simultaneous detection of Escherichia coli O157: H7, toxigenic Vibrio cholerae, and Salmonella typhimurium by multiplex PCR. (2009).

[107] Hoshino K (1998). Development and evaluation of a multiplex PCR assay for rapid detection of toxigenic Vibrio cholerae O1 and O139. FEMS Immunol. Med. Microbiol..

[108] Singh DV (2001). Molecular analysis of Vibrio cholerae O1, O139, non-O1, and non-O139 strains: Clonal relationships between clinical and environmental isolates. Appl. Environ. Microbiol..

[109] Cabrera-García ME, Vázquez-Salinas C, Quiñones-Ramírez EI (2004). Serologic and molecular characterization of Vibrio parahaemolyticus strains isolated from seawater and fish products of the Gulf of Mexico. Appl. Environ. Microbiol..

[110] No Title. *Clin. Lab. Stand. Institute. Perform. Stand. Antimicrob. susceptibility Test. twenty-fifth informational Suppl. CLSI, Wayne, PA, USA CLSI Doc. M100–S25. 2015.*

[111] Osundiya OO, Oladele RO, Oduyebo OO (2013). Multiple antibiotic resistance (MAR) indices of Pseudomonas and Klebsiella species isolates in Lagos University Teaching Hospital. African J. Clin. Exp. Microbiol..

[112] O’Toole GA (1999). Genetic approaches to study of biofilms. Methods Enzymol..

[113] Stepanović S (2007). Quantification of biofilm in microtiter plates: overview of testing conditions and practical recommendations for assessment of biofilm production by staphylococci. APMIS.

