# Supplementary material for: Molecular characterization of Vibrio species isolated from dairy and water samples
Source: Sci Rep. 2023 Sep 16;13:15368. doi: 10.1038/s41598-023-42334-4 (PMC10505214; doi:10.1038/s41598-023-42334-4)
Supplement: Supplementary file 1 — Supplementary Table. [file 41598_2023_42334_MOESM1_ESM.docx]

**Table S1.** Primers sequences, target genes, amplicon sizes and cycling conditions.

| Target agent | Target gene | Primers sequences | Amplified segment (bp) | Primary  denaturation | Amplification (35 cycles) | | | Final extension | Reference |
| --- | --- | --- | --- | --- | --- | --- | --- | --- | --- |
|  |  |  |  |  | Secondary denaturation | Annealing | Extension |  |  |
| *Vibrio spp.* | *16S rDNA* | CGG TGA AAT GCG TAG AGA T | 663 | 94˚C  5 min. | 92°C  40 sec. | 54˚C  30 sec. | 72˚C  40 sec | 72˚C  7 min. | [104] |
|  |  | TTA CTA GCG ATT CCG AGT TC |  |  |  |  |  |  |  |
| *V. parahaemolyticus* | *tox*R | GTCTTCTGACGCAATCGTTG | 368 | 94˚C  5 min. | 94˚C  30 sec. | 60˚C  40 sec. | 72˚C  40 sec. | 72˚C  10 min. | [105] |
|  |  | ATACGAGTGGTTGCTGTCATG |  |  |  |  |  |  |  |
|  | *Trh* | GGCTCAAAATGGTTAAGCG | 250 | 94˚C  5 min. | 94˚C  60 sec. | 55˚C  60 sec. | 72˚C  60 sec. | 72˚C  7 min. | [109] |
|  |  | CATTTCCGCTCTCATATGC |  |  |  |  |  |  |  |
|  | *tdh* | GGTACTAAATGGCTGACATC | 251 | 94˚C  5 min. | 94˚C  30 sec. | 54˚C  40 sec. | 72˚C  40 sec. | 72˚C  7 min. |  |
|  |  | CCACTACCACTCTCATATGC |  |  |  |  |  |  |  |
| *V.cholerae* | *ctx* | ATTTGTTAGGCACGATGATG | 432 | 94˚C  5 min. | 94˚C  30 sec. | 60˚C  40 sec. | 72˚C  45 sec. | 72˚C  10 min. | [106] |
|  |  | ATCGATGATCTTGGAGCATTC |  |  |  |  |  |  |  |
|  | *O1* | GTTTCACTGAACAGATGGG | 192 | 94˚C  5 min. | 94˚C  60 sec. | 55˚C  60 sec. | 72˚C  60 sec. | 72˚C  7 min. | [107] |
|  |  | GGTCATCTGTAAGTA CAAC |  |  |  |  |  |  |  |
|  | *O139* | AGCCTCTTTATTACGGGTGG | 449 |  |  |  |  |  |  |
|  |  | GTCAAACCC GATCGTAAAGG |  |  |  |  |  |  |  |
|  | *ctxAB* | GCCGGGTTGTGGGAATGCTCCAAG | 536 | 94˚C  5 min. | 94˚C  30 sec. | 59˚C  40 sec. | 72˚C  45 sec. | 72˚C  10 min. | [49] |
|  |  | GCCATACTAATTGCGGCAATCGCATG |  |  |  |  |  |  |  |
|  | *tcp*A | CACGATAAGAAAACCGGTCAAGAG | 620 | 94˚C  5 min. | 94˚C  30 sec. | 59˚C  40 sec. | 72˚C  45 sec. | 72˚C  10 min. | [108] |
|  |  | TTACCAAATGCAACGCCGAATG |  |  |  |  |  |  |  |
|  | *hly*A | GGCAAACAGCGAAACAAATACC | 738 | 94˚C  5 min. | 94˚C  30 sec. | 59˚C  40 sec. | 72˚C  60 sec. | 72˚C  10 min. | [108] |
|  |  | CTCAGCGGGCTAATACGGTTTA |  |  |  |  |  |  |  |
